# Detection and identification of mycobacteria in sputum from suspected tuberculosis patients

**DOI:** 10.1186/1756-0500-3-72

**Published:** 2010-03-16

**Authors:** Mochammad Hatta, Andi Rofian Sultan, Nataniel Tandirogang, Masjudicridien_ina@yahoo.co.id, Yadidryadi02@yahoo.com

**Affiliations:** 1 Department of Medical Microbiology, Molecular Biology and Immunology Laboratory for Infectious Diseases, Faculty of Medicine, Hasanuddin University, Jl Perintis Kemerdekaan Km 10 Tamalanrea, Makassar 90245, South Sulawesi, Indonesia

## Abstract

**Background:**

Detection of Tuberculosis agent like nontuberculous mycobacteria (NTM) species by culture and microscopic methods remains difficult and time consuming. A fast and reliable diagnosis of tuberculosis would greatly improve the control of the disease. The purpose of this study is to compare the conventional multiplex PCR and multiplex PCR reverse cross blot hybridization assay to culture method in terms of mycobacteria species detection.

**Findings:**

Among the 117 positively cultured samples, nontuberculous mycobacteria (NTM) species were found in 9 samples of multiplex PCR reverse cross blot hybridization assay; compared to only 3 NTM species found in our conventional multiplex PCR, and 13 NTM species were successfully identified among 162 negatively cultured samples compared to only 5 NTM species identification in conventional multiplex PCR results.

**Conclusions:**

The sensitivity of the multiplex PCR reverse cross blot hybridization assay comparing to culture method was 86.03%, the specificity is 35.46%, the positive predictive value was 41.94% and the negative predictive value was 82.41%. For conventional multiplex PCR these values are 81.62%, 38.65%, 41.89%, 79.51%, respectively. Furthermore, in terms of mycobacteria species detection, the conventional multiplex PCR was relatively equal compared to the multiplex PCR reverse cross blot hybridization assay, and to be particularly having no significant discrepant results on the identification of *Mycobacteria tuberculosis *in both methods.

## Background

Tuberculosis is caused by *Mycobacterium tuberculosis, Mycobacterium bovis or Mycobacterium africanum*. Together with *Mycobacterium microti *and the vaccine strain *M. bovis *BCG they belong to the *Mycobacterium *tuberculosis complex. Some other cases caused by nontuberculous mycobacteria (NTM), are mostly mycobacteria belonging to the *Mycobacterium avium-Mycobacterium intracellulare *complex [1]. Opportunistic mycobacteria commonly associated with the human immunodeficiency virus (HIV) are *Mycobacterium kansasii, Mycobacterium xenopi, Mycobacterium fortuitum and Mycobacterium scrofulaceum *[1]. An estimated 1.7 billion individuals are infected with *Mycobacterium tuberculosis *[2]. Mortality is highest in developing countries, where over three-quarters of cases occur [3].

Early detection is of major importance in the control of tuberculosis [4]. The emergence of multidrug resistant strains and its association with outbreaks on community in endemic areas illustrates that rapid diagnosis is essential [5,6]. A fast and reliable diagnosis of tuberculosis would greatly improve the control of the Tuberculosis [7]. Current conventional diagnosis of tuberculosis or other mycobacteria could be time-consuming, because the culture of mycobacteria may take 4 to 8 weeks. Some mycobacteria are very difficult or almost impossible to grow in vitro such as *M. genavense *and *M. leprae *[4]. Direct staining and microscopy of clinical samples lack sensitivity and specifity [1]. In principle, these drawbacks could be solved by an application of PCR, which allows in vitro amplification of target DNA to a detectable level within a matter of hours [8].

Various researchers have recently described the rapid detection of *M. tuberculosis *by PCR, and many have reported a high degree of sensitivity in detecting *M. tuberculosis *in clinical samples by means of DNA amplifications [8]. Recently a nested PCR has been developed in detecting *Salmonella typhi *in blood, feces and urine from suspect typhoid fever and multiplex PCR to detect *M. tuberculosis *complex bacteria and other mycobacteria which this technique is based on the amplification of the specific insertion sequence IS6110 and 16S rDNA respectively [9,10]. This study uses the multiplex PCR reverse cross blot hybridization assay and the conventional multiplex PCR to detect and identify the Mycobacterium species from clinical samples of patients suspected of mycobacterial diseases in comparison with the Conventional methods.

## Methods

Three hundred and eighty-seven samples of sputum from patients suspected of mycobacterial disease were obtained from the lung hospital in Makassar, Indonesia. Microscopy and culture were performed according to the standard methods at the Department of Medical Microbiology, Molecular Biology and Immunology Laboratory. Ziehl Neelsen staining with some modifications was used for microscopic detection [11]. Sputum samples were decontaminated and cultured on Lowenstein Jensen medium, which is locally produced [12,13], after being extracted with Boom Method, the PCR assays were performed.

### Ethical Approval

This study was reviewed and approved by Hasanuddin University and informed consent was obtained from all participants or their parents or their guardians.

### Multiplex PCR reverse cross blot hybridization

For the amplification of mycobacterial 16S rDNA sequences, the 5'- biotinylated primers pMyc14bio (5'-GAGGTACT CGAGTGGCGAAC-3') and pMyc7bio (5'GGCCGGCTACCCGTCGTC-3') were used. In the PCR mixture, the primer Pt18 (5'GAACCGTGAGGGCATCGAGG-3') and the 5'-biotinylated primer INS2bio (5'-GCGTAGGCGTCGGTGACAAA-3') (Grenner Inc, Japan), were also included, amplifying the *M. tuberculosis *complex-specific insertion sequence IS6110.

Using AmpliTaq Gold PCR Master Mix (AB Applied Biosystem, USA), samples were incubated for 10 minutes at 40°C, to break down possible contaminating amplicons by Uracil DNA glycosylase (UDG) [12,14]. Then, incubated at 94°C for 40 seconds, 65°C for 40 seconds and 50 seconds at 72°C, with 40 cycles.

### Tailing of oligonucleotide probes with dTTP

The tailing reactions were performed with 200 pmol of the oligonucleotide probe. The probes were fixed to the membrane in a hybridization oven for 10 minutes. The membrane was washed twice with 10× SSC. The probes chosen for the identification of 16S rDNA and IS6110 PCR products (Table 1). On the rotary shaker for at least 5 minutes, the membrane was put in the cross blotter on the rubber mat, and a different mould with 34 slots [2 × 50 mm (numbered 0-33)] or a mould with three blocks of 34 slots (each 2 × 15 mm) was placed on top of it. The hybridized PCR product on the membrane was detected by incubation with streptavidin-alkaline phosphatase and a color substrate (4-nitroblue tetrazolium chloride and 5-bromo-4-chloro-3-indolylphosphate) according to the instruction of the manufacturer (Boehringer Mannheim, Germany).

**Table 1:** Multiplex PCR reverse cross blot hybridization results

PCR Probe	Mycobacterium Species		Cultures	
			(+)ve	(-)ve
pMyc5a	*Mycobacterium spp*	5'-GGGCCCATCCCACACCGC-3'	117	162
pAvi7	*M. avium*	5'CCAGAAGACATGCGTCTTGAG-3'	3	5
pInt5	*M. intracellulare*	5'-CACCTAAAGACATGCGCCTAA-3'	1	1
pInt7	*M. intracellulare*	5'-CACCAAAAGACATGCGTCTAA-3'	1	1
pKan7	*M. kansasii*	5'CAAGGCATGCGCCAAGTGGT-3'	1	1
pXen1	*M. xenopi*	5'-ACCACCCCACATGCGGAGAA-3'	0	0
pFor1	*M. fortuitum*	5'-ACCACACACCATGAAGCGCG-3'	1	1
pChe3	*M. chelonae*	5'-CCACTCACCATGAAGTGTGTG-3'	2	3
pGen1	*M. genavense*	5'-CCACAAAACATGCGTTCCGTG-3'	0	1
pGor5	*M. gordonae*	5'-TGTGTCCTGTGGTCCTATTCG-3	0	0
pMar2	*M. marinum*	5'-CGGGATTCATGTCCTGTGGT-3'	0	0
Pt3	*M. tuberculosis complex*	5'-GAACGGCTGATGACCAAACT-3'	117	162
pSme3	*M. smegmatis*	5'-CATGCGACCAGCAGGGTGTA-3'	1	1
pTub1	*M. tuberculosis complex*	5'-AACACAAGACATGCATCCCG-3'	108	149

### Conventional multiplex PCR [15]

Primers (HT1: 5'-CCTGCGAGCGTAGGCGTCGG-3'; HT2: 5'-CTCGTCCAGC GCCGCTTCGG-3'; HT3: 5'-CTTGCTGGAGGTGCTCGACG-3'and HT4: 5'-GGAGGTGCCGT GCAGGTAGG-3') 0.5 uM of each, 5 ul DNA template and 47 ul of distilled water (Ultrapure, Invitrogen Co, Japan) were added to a 0.2 microcentrifuge tube containing AmpliTaq Gold. Conditions for thermocycling were as follow: 95°C for 10 minutes, 40 cycles of amplification (94°C for 30 seconds followed by 60°C for 40 seconds and 72°C for 40 seconds) and 72°C for 10 minutes. Using 1.8% agarose gel containing ethidium bromide (Sigma, USA), 5 uL of PCR product were analysed by electrophoresis at 100 V for 30 minutes. PCR Product length for HT1/HT2 and HT3/HT4 are 123 base pairs (bp) for *M. Tuberculosis *and 322 bp for *M. avium*.

### Statistical analysis

Difference in the results between positive and negative groups for culture, microscopy, and conventional multiplex PCR in the same sputum samples and for multiplex PCR reverse cross blot hybridization assay results were analyzed using the SPSS (SPSS Inc., Chicago, Il) computer package.

## Findings

### The electrophoresis of conventional multiplex PCR and pattern of multiplex PCR reverse cross blot hybridization in nitrocellulose membrane

**Figure 1:**
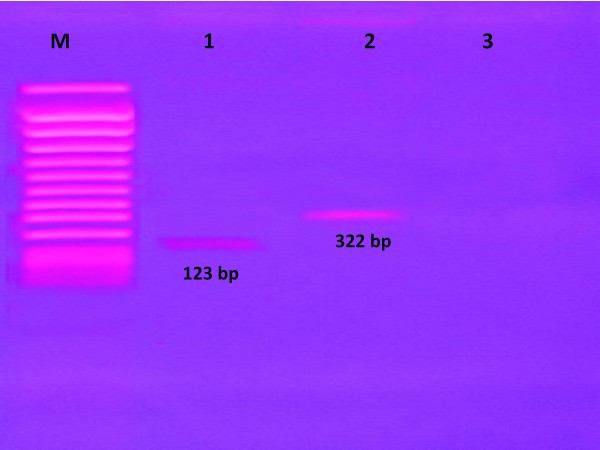
**Gel electrophoresis of conventional multiplex PCR result**. M, molecular weight Marker; line 1, *M. tuberculosis*; line 2, *M. avium*; line 3, *M. chelonae*.

The conventional multiplex PCR was set by specific primers to determine the insertion sequences of IS6110 and IS1245. Figure 1. shows representative of the DNA amplified products by conventional multiplex PCR and these amplicons of PCR product which were analyzed by electrophoresis on 1.8% agarose gels stained with ethidium bromide (Sigma, USA), 100 V for 30 minutes and the result was recorded by photography camera under ultraviolet light. *M. tuberculosis *with 123 bp PCR product (line 1) and *M. avium *with 322 bp PCR product (line 2), but *M. chelonae *was not detected in this conventional multiplex PCR method (line 3).

**Figure 2:**
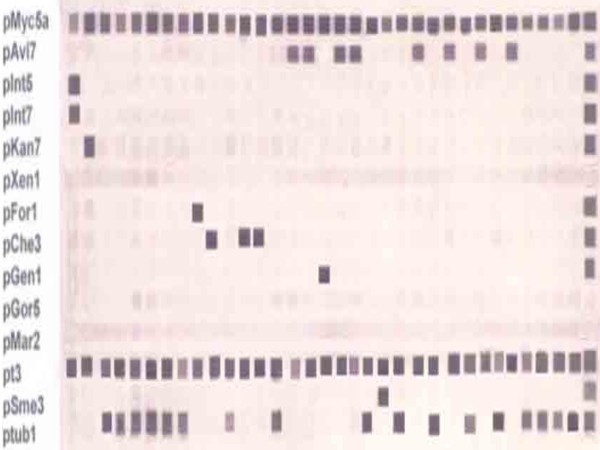
**Multiplex PCR reverse cross blot hybridization assay result**. Line 1, *M. intracellulare*; line 2, *M. kansasii *; line 3-8, 11, 14, 20, 22, 24, 26, 28, 30-33, *M. tuberculosis*; line 9, *M. fortuitum*; line 10, 12, 13, *M. chelonae*; line 15, 16, 18, 19, 23, 25, 27, 29, *M. avium*; line 17, *M. genavense*; line 21, *M. smegmatis *; 34, pool PCR product of mycobacteria.

Figure 2. shows representative of the multiplex PCR reverse cross blot hybridization results in nitrocellulose membrane. All samples were shown positive hybridized to pMyc5a and pt3 probes. *M. intracellare *was shown positive hybridized to pInt5 and pInt7 (line 1) and *M. kansasii*, *M. tuberculosis*, *M. fortuitum*, *M. cholenae*, *M. avium*, *M. genavense*, *M. smegmatis *was shown positive hybridized to pKan7 (line 2), ptub1 (line 3-8, 11, 14, 20, 22, 24, 26, 28, 30-33), pFor1 (line 9), pChen3 (line 10, 12, 13), pAvi7 (line 15, 16, 18, 19, 23, 25, 27, 29), pGen1 (line 17), pSme3 (line 21), respectively. In positive control, the amplicons of mixed PCR product of mycobacteria was used (line 34).

### The culture, microscopy and multiplex PCR reverse cross blot hybridization assay

The culture positive samples were divided into 117 positive and 19 negative multiplex PCR reverse cross blot hybridization results (table 2). From the 117 positive in culture samples, 108 were also positive in PCR reverse cross blot hybridization assay (for the probes pMyc5a, pt3 and pTub1 9 samples were found negative for the probe pMyc5a and pPt3) and 3 were positive pAvi7; 2 samples were positive pChe3 and 1 sample was positive in the probe pInt5, pInt7, pKan7, pFor1, pSme3, respectively (table 1). From the 251 negative culture results 162 were positive in multiplex PCR reverse cross blot hybridization assay (table 2). In 162 positive in multiplex PCR reverse cross blot hybridization assay, 149 samples gave positive results with the probes pMyc5a, pt3 and pTub1 except 13 samples were found negative for the probe pMyc5a and pPt3 and the other 5 samples were positive with the probe pAvi7; 3 samples gave positive result in pChe3 and 1 sample was positive in the probe pInt5, pInt7, pKan7, pFor1, pSme3, respectively (table 1). Sputum samples with microscopy positive were found also positive in sputum cultures and no significant statistical difference exists between microscopy and culture tests (p > 0.05) (table 2). The sensitivity of the Multiplex PCR reverse cross blot hybridization assay compared to culture method was 86.03%, the specificity was 35.46%, the positive predictive value was 41.94% and the negative predictive value was 82.41%.

**Table 2:** Comparison of culture, microscopy and multiplex PCR reverse cross blot hybridization assay

PCR Results	Number of samples			
	with culture results		Microscopy results	
	Positive (n = 136)	Negative (n = 251)	Positive (n = 115)	Negative (n = 272)
Positive	117	162	102	177
Negative	19	89	13	95

### Comparison between conventional multiplex PCR and multiplex PCR reverse cross blot hybridization assay results

From 117 positive cultured samples which then amplified with multiplex PCR reverse cross blot hybridization assay, it was found that 108 samples were *M. tuberculosis *and 3 samples of *M. avium*. These findings were similar with the conventional multiplex PCR results. The rest 6 samples were identified as the followings: 2 samples positive for *M. chelonae *and 1 sample showed positive *M. intracellulare*, *M. kansasii*, *M. fortuitum *and *M. smegmatis*, respectively. However, these 6 last mentioned samples were not identified by the conventional multiplex PCR assay (table 3).

**Table 3:** Comparison of conventional multiplex PCR and PCR reverse cross blot hybridization assay

	Conventional multiplex PCR positive/PCR Cross Blot Hybridization positive	
	Positive Culture (n = 136)	Negative culture (n = 251)
*M. Tuberculosis*	108/108	149/149
*M. avium*	3/3	5/5
*M. intracellulare*	0/1	0/1
*M. kansasii*	0/1	0/1
*M. fortuitum*	0/1	0/1
*M. chelonae*	0/2	0/3
*M. genavense*	0/0	0/1
*M. smegmatis*	0/1	0/1
No amplification	25/19	97/89

Moreover, among 162 positive samples by multiplex PCR reverse cross blot hybridization but negative in culture, 149 samples were positive for *M. tuberculosis *and 5 samples of *M. avium*. These findings again showed similarity with the conventional multiplex PCR results. Other 3 samples positive for *M. chelonae *and 1 sample was shown positive *M. intracellulare*, *M. kansasii*, *M. fortuitum*, *M. genavense *and *M. smegmatis*, respectively. These last mentioned 8 samples were failed the conventional multiplex PCR detection. (tables 3). The sensitivity of the conventional Multiplex PCR comparing to culture method was 81.62%, the specificity was 38.65%, the positive predictive value was 41.89% and the negative predictive value was 79.51%. No significant difference was found in identification of *M. tuberculosis *by conventional multiplex PCR and multiplex PCR reverse cross blot hybridization assay results in both culture positive and negative samples.

## Discussion

The results of this study show that multiplex PCR and reverse cross blot hybridization significantly are more sensitive than culture and microscopic methods to detect mycobacteria strain (p < 0.05) (table 1). The sensitivity of the Multiplex PCR Reverse cross blot hybridization assay comparing to culture method was 86.03%, the specificity was 35.46%, the positive predictive value was 41.94% and the negative predictive value was 82.41%. For Conventional Multiplex PCR these values were 81.62%, 38.65%, 41.89%, 79.51% respectively (table 4). Furthermore, no significant difference was found on the identification of *M. tuberculosis *by conventional multiplex PCR and multiplex PCR reverse cross blot hybridization assay results in both positive and negative samples of culture results. The low specificity of both PCR assays was presumably due to the higher positive result among negative culture of samples. This could be resulting from a high number of samples with fastidious or non cultivable mycobacteria content such as *M. genavense *and *M. leprae*, and also there was an evidence that some of the samples were positive in culture but negative on both of our PCR methods, this probably due to contamination of other bacteria that easily happened in culture methods.

**Table 4:** The Sensitivity and the Specificity of conventional multiplex PCR and PCR reverse cross blot hybridization results

	Multiplex PCR	
	PCR Konvensional	Reverse Cross blot Hybridization
	(%)	(%)
Sensitivity	81.62	86.03
Specifity	38.65	35.46
Positive Predictive Value	41.89	41.94
Negative Predictive value	79.51	82.41

In terms of mycobacteria species detection, the conventional multiplex PCR was relatively equal compared to the multiplex PCR reverse cross blot hybridization. Conventional multiplex PCR method is easier and simpler in application compared to multiplex PCR reverse blot cross hybridization assay. On the other hand, Multiplex PCR reverse cross blot hybridization is a more complicated method; however it can detect considerably more nontuberculous mycobacteria (NTM) species such as *M. avium*, *M. intracellulare*, *M. kansasii*, *M. fortuitum*, *M. chelonae*, *M. genavense* and *M. smegmatis*, something that is unidentifiable by our conventional Multiplex PCR.

The conventional multiplex PCR and Multiplex PCR reverse cross blot hybridization assay should be suitable for a rapid and correct diagnosis of patients suspected of having mycobacterial disease. These two methods will help the clinicians significantly in deciding the suitable antimicrobial treatment for their patients within shorter period of time.

## Competing interests

The authors declare that they have no competing interests.

## Authors' contributions

MH carried out the molecular studies, drafted the manuscript and performed the statistical analysis; ARS carried out the culture and participated in PCR test; NT, M and Y helped to collect isolates, participated in the design of the study and helped to perform statistical analysis. All authors read and approved the final manuscript.
